# Endoscopic management of a chronic anastomotic leak after a Billroth II procedure

**DOI:** 10.1055/a-2228-4533

**Published:** 2024-01-30

**Authors:** Reid D. Wasserman, William F. Abel, Arnold Salzberg, Vivek Kesar, Paul Yeaton, Varun Kesar

**Affiliations:** 1 Internal Medicine, Virginia Tech Carilion Clinic, Roanoke, United States; 2 Surgery, Virginia Tech Carilion Clinic, Roanoke, United States; 3 Gastroenterology and Hepatology, Virginia Tech Carilion Clinic, Roanoke, United States

We report the case of a 56-year-old woman with a pertinent past medical history of chronic pancreatitis and peptic ulcer disease, who initially presented with a perforated posterior duodenal ulcer following a Billroth II procedure, which was complicated by a duodenal stump abscess and purulent free fluid in the pelvis. The patient subsequently underwent a computed tomography (CT) scan of the abdomen, which revealed extraluminal contrast extravasation within the left upper quadrant, raising concern of an anastomotic leak ( Fig. 1 ). The patient subsequently underwent upper gastrointestinal endoscopy with argon plasma coagulation (APC) and full-thickness endoscopic suturing, with placement of a nasojejunal tube (NJT) in the efferent jejunal limb ( Fig. 2 ; Video 1 ). An upper gastrointestinal series was performed the next day, which showed no evidence of residual leakage ( Fig. 3 ). The patient was discharged with instructions to remain nil by mouth, and an NJT for tube feeding, along with oral antibiotics. Subsequently, she was started on an oral diet and tolerated this without any complications. At follow-up, 3 months after the procedure, the patient was tolerating solid food well.

**Fig. 1:**
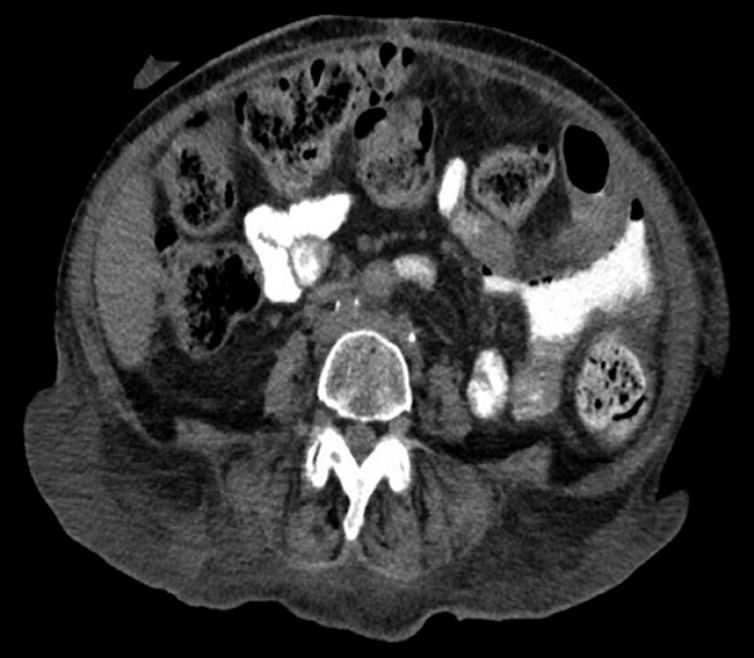
Computed tomography image of the abdomen showing extraluminal contrast extravasation within the left upper quadrant, raising concern of an anastomotic leak.

**Fig. 2:**
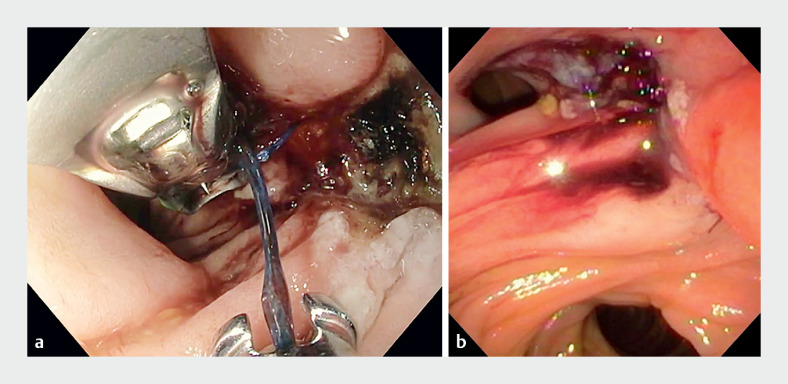
Endoscopic views showing the anastomotic leak site: **a** during suturing; **b** after endoscopic suturing.

**Fig. 3:**
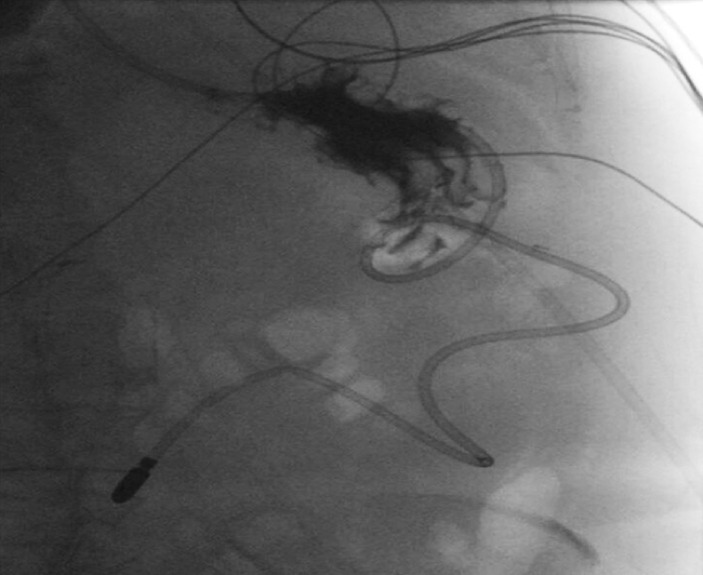
Radiographic image from an upper gastrointestinal series showing no evidence of contrast extravasation, indicating no leakage, and the nasojejunal tube in the efferent limb *.*

Upper gastrointestinal endoscopy of a chronic anastomotic leak being treated with argon plasma coagulation (APC) and full-thickness endoscopic suturing, with a subsequent upper gastrointestinal series showing no evidence of residual leakage.Video 1

Billroth II (antrectomy with gastrojejunostomy) serves as a surgical management option for perforated gastric ulcers. Anastomotic leakage is a serious complication of Billroth II surgery and occurs 7–10 days after surgery. Surgical intervention is the mainstay of treatment, but may not be amenable for patients who are high risk for morbidity and mortality 1 . While a wide variety of endoscopic therapies exist for the management of leaks, endoscopic suturing has shown clinical success in retrospective studies 1 . This case demonstrates the successful repair of a chronic anastomotic Billroth II leak using APC and endoscopic suturing. Endoscopic suturing is an effective and alternative intervention that can be used for high risk patients who are unable to undergo traditional surgical intervention.

Endoscopy_UCTN_Code_TTT_1AO_2AI

## Notes

Endoscopy E-Videos https://eref.thieme.de/e-videos
*E-Videos* is an open access online section of the journal *Endoscopy* , reporting on interesting cases and new techniques in gastroenterological endoscopy. All papers include a high-quality video and are published with a Creative Commons CC-BY license. Endoscopy E-Videos qualify for HINARI discounts and waivers and eligibility is automatically checked during the submission process. We grant 100% waivers to articles whose corresponding authors are based in Group A countries and 50% waivers to those who are based in Group B countries as classified by Research4Life (see: https://www.research4life.org/access/eligibility/ ). This section has its own submission website at https://mc.manuscriptcentral.com/e-videos .
